# Activation of a non-neuronal cholinergic system in visceral white adipose tissue of obese mice and humans

**DOI:** 10.1016/j.molmet.2023.101862

**Published:** 2023-12-22

**Authors:** Ilenia Severi, Jessica Perugini, Chiara Ruocco, Lara Coppi, Silvia Pedretti, Eleonora Di Mercurio, Martina Senzacqua, Maurizio Ragni, Gabriele Imperato, Alessandra Valerio, Nico Mitro, Maurizio Crestani, Enzo Nisoli, Antonio Giordano

**Affiliations:** 1 Department of Experimental and Clinical Medicine, Marche Polytechnic University, 60126 Ancona, Italy; 2 Center for Study and Research on Obesity, Department of Medical Biotechnology and Translational Medicine, University of Milan, 20129 Milano, Italy; 3 Department of Pharmacological and Biomolecular Sciences, University of Milan, 20122 Milano, Italy; 4 Department of Molecular and Translational Medicine, University of Brescia, 25123 Brescia, Italy; 5 Department of Experimental Oncology, IEO, European Institute of Oncology IRCCS, Milano, Italy; 6 Center of Obesity, Marche Polytechnic University-United Hospitals, Ancona, Italy

**Keywords:** Acetylcholine, Obesity, Insulin resistance, Choline, Inflammation, Macrophages

## Abstract

**Background and objectives:**

Since white adipose tissue (WAT) lacks parasympathetic cholinergic innervation, the source of the acetylcholine (ACh) acting on white adipocyte cholinergic receptors is unknown. This study was designed to identify ACh-producing cells in mouse and human visceral WAT and to determine whether a non-neuronal cholinergic system becomes activated in obese inflamed WAT.

**Methods:**

Mouse epididymal WAT (eWAT) and human omental fat were studied in normal and obese subjects. The expression of the key molecules involved in cholinergic signaling was evaluated by qRT-PCR and western blotting whereas their tissue distribution and cellular localization were investigated by immunohistochemistry, confocal microscopy and *in situ* hybridization. ACh levels were measured by liquid chromatography/tandem mass spectrometry. The cellular effects of ACh were assessed in cultured human multipotent adipose-derived stem cell (hMADS) adipocytes.

**Results:**

In mouse eWAT, diet-induced obesity modulated the expression of key cholinergic molecular components and, especially, raised the expression of choline acetyltransferase (ChAT), the ACh-synthesizing enzyme, which was chiefly detected in interstitial macrophages, in macrophages forming crown-like structures (CLSs), and in multinucleated giant cells (MGCs). The stromal vascular fraction of obese mouse eWAT contained significantly higher ACh and choline levels than that of control mice. ChAT was undetectable in omental fat from healthy subjects, whereas it was expressed in a number of interstitial macrophages, CLSs, and MGCs from some obese individuals. In hMADS adipocytes stressed with tumor necrosis factor α, ACh, alone or combined with rivastigmine, significantly blunted monocyte chemoattractant protein 1 and interleukin 6 expression, it partially but significantly, restored adiponectin and GLUT4 expression, and promoted glucose uptake.

**Conclusions:**

In mouse and human visceral WAT, obesity induces activation of a macrophage-dependent non-neuronal cholinergic system that is capable of exerting anti-inflammatory and insulin-sensitizing effects on white adipocytes.

## Highlights

• Visceral fat macrophages synthesize acetylcholine (ACh).
• Under obesity, a non-neuronal cholinergic system is activated in visceral fat.
• Cholinergic signalling is promoted in both mouse and human obese visceral fat.
• ACh reduces pro-inflammatory cytokine expression in cultured human adipocytes.
• ACh increases GLUT4 expression and glucose uptake in cultured human adipocytes.

## 1 Introduction

Obesity, characterized by a massive increase of white adipose tissue (WAT) in the body, is a major and growing public health problem that involves a significantly increased risk of developing several chronic and life-threatening comorbidities including type 2 diabetes, cardiovascular and liver disease, certain cancers, and neurodegeneration [[1], [2], [3]]. Well-established clinical evidence shows that obesity-associated comorbidities mostly develop in subjects with visceral obesity, where intraperitoneal WAT expands in proximity and close functional relationship to abdominal organs and structures [4,5].

In animal models of obesity and in human obesity, visceral WAT shows early activation of a low-grade, chronic inflammatory program [6,7]. Accordingly, visceral adipose tissue displays prominent metabolic abnormalities including adipocyte hypertrophy and stress; accelerated adipocyte death rate; infiltration by inflammatory cells, mainly macrophages; increased secretion of free fatty acids and proinflammatory mediators; and adipokine and exosome dysregulation [[8], [9], [10], [11]]. On histology, macrophages distinctively cluster around dead adipocytes – forming the so-called crown-like structures (CLSs) – where aggregates of activated macrophages, sometimes fused into syncytia (multinucleated giant cells, MGCs), clear the remnants of dead adipocytes. Over time, inflamed and dysfunctional visceral WAT develops increasingly marked alterations of the vascular supply and fibrosis [12].

In mammals, white adipocytes express muscarinic (mAChRs) [13] and, especially, nicotinic cholinergic receptors (nAChRs) [[14], [15], [16], [17], [18], [19]]. mAChRs are metabotropic receptors, of which five subtypes (M1-M5) have been identified: M1, M3 and M5 subtypes are coupled to the phosphoinositide signaling pathway, whereas M2 and M4 are linked to the adenylate cyclase system [20]. In contrast, nAChRs are ionotropic receptors that are composed of five distinct subunits, which form a ligand-gated ion channel allowing rapid and transient translocation across the cell membrane of monovalent (sodium and potassium) or bivalent (calcium) ions [21]. Activation of adipocyte nAChRs by nicotine or isotype-specific agonists is associated with beneficial metabolic and anti-inflammatory effects, which have been described both in obese fat and in cultured rodent and human white adipocytes [[14], [15], [16], [17], [18], [19]]. Specifically, in genetically (*db*/*db*) and diet-induced obese mice, α7nAChR activation by nicotine, at doses not involving changes in food intake or body weight, reduces adipose tissue inflammation and improves body glucose homeostasis and insulin sensitivity [22], whereas activation of α2nAChR in mouse subcutaneous fat promotes the development of energy-dissipating beige adipocytes, increases non-shivering thermogenesis, and ameliorates diet-induced obesity [23].

A major and still unanswered question is the origin, or cellular source, of acetylcholine (ACh), which is capable of reaching white adipocytes and of interacting with their cholinergic receptors, thus affecting adipocyte biology in both physiological and pathological conditions. ACh is an extensively studied neurotransmitter with a wide distribution. It is synthesized from choline and acetyl-coenzyme A by choline acetyltransferase (ChAT) in the cytosol of several classes of central and peripheral neurons. Subsequently, it is stored in synaptic vesicles by the vesicular ACh transporter (VAChT). When the action potential reaches the cholinergic axon terminal, the vesicles fuse with the presynaptic membrane, ACh is secreted in the synaptic cleft and quickly reaches the postsynaptic membrane, where it interacts with its receptors. ACh released by nerve endings is effectively and rapidly cleared by acetylcholinesterase (AChE) and butyrylcholinesterase (BuChE), membrane-bound and/or extracellular matrix-inserted enzymes that hydrolyze ACh to acetate and choline [24]. Choline, an essential nutrient of mammalian cells [25], is quickly taken up from the extracellular space by neurons and effector cells through choline transporter 1 (ChT1), to be reused for ACh synthesis or other metabolic needs. In the periphery, most parasympathetic postganglionic neurons are cholinergic. However, since subcutaneous and visceral WAT both lack parasympathetic cholinergic innervation [26], a neural origin of the ACh acting on white adipocytes is unlikely.

Over the past few decades, a mounting body of evidence has shown that the molecular components required for cholinergic neurotransmission are also found in organs devoid of innervation (typically the placenta) or lacking cholinergic nerves (typically the spleen), and that besides neuronal cells they are also expressed by epithelial, mesothelial, endothelial, circulating and immune cells [[27], [28], [29]]. In brief, mammals, in addition to neuronal cholinergic systems, also possess non-neuronal cholinergic systems, where secreted ACh is involved in cell–cell communication and controls basic cell functions including mitosis, differentiation, cytoskeleton organization, cell–cell contact, secretion and absorption [[27], [28], [29]].

In several inflammatory diseases, granulocytes, lymphocytes and macrophages acquire the ability to synthesize and secrete ACh, which regulates immune responses and mediates anti-inflammatory effects through auto- and/or paracrine loops [30,31]. Thus, we decided to investigate whether a non-neuronal cholinergic system is present in WAT and becomes activated in obesity-induced WAT inflammation, acting on obese white adipocytes. Collectively, our results show that in obesogenic conditions mouse and human visceral WAT both contain a significant number of infiltrating cells, mainly macrophages, capable of synthesizing and secreting ACh, which in turn drives adipocyte metabolism toward a less inflammatory and more insulin-sensitive phenotype.

## 2 Methods

### 2.1 Animals and tissues

Male C57BL/6 mice were purchased from Charles River (Lecco, Italy) and housed in plastic cages under constant environmental conditions (12 h light/dark cycle at 22 °C) with *ad libitum* access to food and water. Animal care was according to Council Directive 2010/63/EU. All experiments were approved by the Italian Health Ministry (authorization no. 405/2018-PR). Animals initially received a standard chow diet (CD; Envigo, Indianapolis, IN; 24 kJ% from protein, 18 kJ% from fat, 58 kJ% from carbohydrates). At age 5 weeks, some mice were switched to a high-fat diet (HFD; Research Diets, New Brunswick, NJ; 20 kJ% from protein, 60 kJ% from fat, 20 kJ% from carbohydrates) for 15 weeks. All experiments were carried out in mice aged 20-22-weeks. For qRT-PCR and western blotting, anesthetized animals were decapitated, epididymal WAT (eWAT) was dissected out, snap-frozen in liquid nitrogen and stored at −80 °C. For liquid chromatography/tandem mass spectrometry (LC-MS-MS) analysis, eWAT was processed immediately. For morphological studies, anesthetized animals were perfused transcardially with 4% paraformaldehyde in 0.1 M phosphate buffer (PB), pH 7.4, eWAT was isolated using a Zeiss OPI1 surgical microscope (Carl Zeiss, Oberkochen, Germany) and further fixed by immersion in 4% paraformaldehyde in PB overnight at 4 °C. After a thorough rinse in PB, specimens were dehydrated in ethanol, cleared in xylene, and embedded in paraffin.

The human samples were omental WAT biopsies from 31 patients with severe obesity (body mass index [BMI] ≥ 35 kg/m^2^) and from 10 control subjects (BMI ≤ 30 kg/m^2^) undergoing respectively bariatric surgery and cholecystectomy at Ancona General Hospital-Azienda Ospedaliera Universitaria (AOU, Ospedali Riuniti, Ancona, Italy). Each biopsy was divided into two halves: samples for qRT-PCR were snap-frozen in liquid nitrogen and stored at −80 °C, whereas those for morphological analyses were fixed in formalin for 24 h at 4 °C and paraffin-embedded. The study protocol was approved by the Ethics Board of Ospedali Riuniti (Ancona, Italy). Subjects receiving anti-inflammatory drugs were excluded from the study.

### 2.2 Cell culture and treatments

The cell culture media, fetal bovine serum (FBS), buffers, and trypsin were from Pan-Biotech GmbH (Aidenbach, Germany); the cell culture reagents, including Oil Red O, ACh, rivastigmine, and insulin were obtained from Sigma–Aldrich (Milano, Italy). Human recombinant fibroblast growth factor (hFGF)-2 and human recombinant tumor necrosis factor α (TNFα) were purchased from PeproTech (London, UK). Human multipotent adipose-derived stem cells (hMADS), kindly provided by Dr Christian Dani (Université Côte D'Azur, Nice, France) were cultured as described previously [32,33]. In brief, hMADS grown in low-glucose (1 g/L) proliferation medium (Dulbecco's modified Eagle's medium [DMEM]) supplemented with 10% FBS and 2.5 ng/ml hFGF-2 were used between the 16th and the 19th passage. To induce adipose differentiation, they were seeded in proliferation medium on multi-well plates at a density of 4500 cells/cm^2^. When they reached confluence hFGF-2 was not replaced. The next day (day 0), cells were incubated in an adipogenic medium (serum-free proliferation medium/Ham's F-12 medium) containing 10 μg/ml transferrin, 5 μg/ml insulin, 0.2 nM triiodothyronine, 100 μM 3-isobutyl-1-methylxanthine, 1 μM dexamethasone, and 100 nM rosiglitazone. Dexamethasone and 3-isobutyl-1-methylxanthine were not replaced from day 3 and rosiglitazone from day 9. Cell lipid content was assessed at different time points by Oil Red O staining. Treatments and biological assays were carried out on differentiated hMADS adipocytes from day 12 to day 15. Treatment duration and ACh, rivastigmine, TNFα and insulin concentrations are reported in the Results section.

### 2.3 qRT-PCR

Total RNA was extracted with TRIZOL reagent (Invitrogen, Carlsbad, CA), purified, digested with ribonuclease-free deoxyribonuclease, and concentrated using RNeasy Micro kit (Qiagen, Milano, Italy) according to the manufacturer's instructions. For determination of mRNA levels, 1 μg RNA was reverse-transcribed with High-Capacity cDNA RT Kit with RNase Inhibitor (Applied BioSystems, Foster City, CA) in a total volume of 20 μl. qRT-PCR was performed using TaqMan Gene Expression Assays and Master Mix TaqMan (Applied BioSystems). All probes were from Applied BioSystems (Suppl. Table 1A). Reactions were carried out in a Step One Plus Real Time PCR system (Applied BioSystems) using 50 ng cDNA in a final reaction volume of 10 μl. The thermal cycle protocol included initial incubation at 95 °C for 10 min followed by 40 cycles of 95 °C for 15 s and of 60 °C for 20 s. A control reaction without reverse transcriptase in the amplification mixture was performed for each sample to rule out genomic contamination. Samples not containing the template were included in all experiments as negative controls. TATA box-binding protein was used as an endogenous control to normalize gene expression. Relative mRNA expression was determined by the ΔCt method (2^−ΔCt^).

### 2.4 Glucose uptake

hMADS adipocytes were differentiated in 96-well black-bottom plates (Corning, Sigma–Aldrich) and incubated in low-glucose DMEM with 1 μM ACh and 100 nM rivastigmine for different periods of time, as reported in the Results. Next, adipocytes were incubated with or without 100 nM insulin in Krebs–Ringer PB (pH 7.4) at 37 °C for 10 min. They were then treated with 2-nitrobenzodeoxyglucose (50 μM) for 60 min. Fluorescence intensity was evaluated at 550/590 nm using an Infinite F200 PRO plate reader (Tecan, Mannedorf, Switzerland).

### 2.5 Cytokine release measurement

Cytokine release was measured by ELISA in conditioned media collected at the end of each incubation. The media were subsequently centrifuged and stored at −80 °C until use. Interleukin (IL)-6 (pg/ml) and adiponectin (ng/ml) concentrations were measured according to the manufacturer's instructions using respectively Human Adiponectin ELISA kit (# AG-45A-0001YEK-KI01, Adipogen Life Sciences, Switzerland) and Human IL-6 ELISA kit (# 501030, Cayman Chemical, Ann Arbor, MI).

### 2.6 Western blotting

Protein extracts were obtained from eWAT using the Tissue Protein Extraction Reagent (T-PER, Pierce, Thermo Scientific, Rockford, IL), as indicated by the manufacturer, after adding the protease and phosphatase inhibitor cocktail (Sigma–Aldrich). Protein content was determined with the bicinchoninic acid protein assay (Euroclone, Milano, Italy). An appropriate amount of protein was run on sodium dodecyl sulfate–polyacrylamide gel electrophoresis (SDS-PAGE) under reducing conditions for immunoblotting. The separated proteins were then semi-dry-transferred to a nitrocellulose membrane (Bio-Rad, Milano, Italy) and proteins of interest were detected with specific antibodies (Suppl. Table 1B). Anti-vinculin was used as a loading control. When the membranes were used for detection of more than one protein, filters were stripped with Euroclone Restore Western Blot Stripping Buffer and then incubated with the primary antibody as suggested by the manufacturer. Immunostaining was detected using horseradish peroxidase conjugated anti-rabbit or anti-mouse immunoglobulin (Suppl. Table 1C) [34]. The amounts of each protein were measured using SuperSignal Substrate (Euroclone), analyzed with Chemidoc XRS+ and quantified by ImageLab software (both from Bio-Rad).

### 2.7 Peroxidase immunohistochemistry, confocal microscopy and morphometric analysis

Peroxidase immunohistochemistry was performed on 3-μm-thick paraffin sections. Sections were dewaxed, washed in 0.1% Tween-phosphate buffered saline (PBS) and incubated with an antigen retrieval solution (Histo-VT-One, PH 9, Nacalai Tesque, Kyoto, Japan) for 40 min at 70 °C. After a thorough rinse in PBS, sections were reacted with 0.3% H_2_O_2_ (in PBS; 30 min) to block endogenous peroxidase, rinsed with PBS and incubated in blocking solution (3% normal serum in PBS; 60 min). Next, they were incubated with the primary antibody (Suppl. Table 1B; in PBS; overnight at 4 °C). After a thorough rinse in PBS, sections were incubated in a 1:200 v/v biotinylated HRP-conjugated secondary antibody solution (Suppl. Table 1C; in PBS; 30 min, at room temperature [RT]). Histochemical reactions were performed using Vectastain ABC Kit (Vector Laboratories, Burlingame, CA) and Sigma Fast 3,3′-diaminobenzidine (Sigma–Aldrich) as the substrate. Sections were finally counterstained with hematoxylin, dehydrated and mounted in Eukitt (Sigma–Aldrich). Staining was never observed when the primary antibody was omitted.

For immunofluorescence and multiple-labeling experiments, sections were processed as described above up to and including incubation with the primary antibodies (Suppl. Table 1B). The next day sections were washed twice with PBS and incubated with a mixture of secondary antibodies diluted in 1% bovine serum albumin-PBS (Suppl. Table 1C) 30 min at RT. Sections were subsequently washed twice with 0.1% Tween-PBS, stained with TO-PRO3 (T3605, Invitrogen, Carlsbad, CA), mounted, and coverslipped using Vectashield mounting medium (Vector Laboratories). Sections were viewed under a motorized DM6000 microscope (Leica Microsystems, Wetzlar, Germany) at different magnifications. Fluorescence was detected with a Leica TCS-SL spectral confocal microscope equipped with an Argon and He/Ne mixed gas laser. Fluorophores were excited with the 488, 543 and 649 nm lines and imaged separately. Images (1024 × 1024 pixels) were obtained sequentially from three channels using a confocal pinhole of 1.1200 and stored as TIFF files. The brightness and contrast of the final images were adjusted using Photoshop 6 (Adobe Systems). The percentage of F4-80-positive interstitial macrophages, CLS macrophages and MGCs also expressing ChAT and the percentage of ChAT-positive interstitial cells, CLS macrophages, and MGCs also expressing CD206 or CD11c were calculated in 5 mice *per* experimental group. The percentage of CD68-positive interstitial macrophages, CLS macrophages, and MGCs also expressing ChAT was calculated in omental fat sections from 7 obese subjects. For both mouse and human morphometry, three representative 50-μm-spaced sections from each sample were analyzed; from each section, 10 non-overlapping images were randomly selected using a × 63 objective.

### 2.8 RNA *in situ* hybridization

Paraffin-embedded sections 5 μm in thickness were mounted on SuperFrost Plus slides (Thermo Fisher, Waltham, MA), dried at RT overnight, baked at 60 °C for 1 h and deparaffinized. RNA *in situ* hybridization (FISH RNAscope® technology; ACDBio, Bio-Techne, Minneapolis, MN) was performed using specific RNA probes (Suppl. Table 1D) according to the manufacturer's instructions. Then, 3-plex negative and 3-plex positive control probes were processed in parallel with target probes. All incubation steps were performed at 40 °C using the ACD HybEz hybridization system (ACDBio). Sections were subsequently incubated with H_2_O_2_ at RT for 10 min, rinsed twice in distilled water and subsequently submerged in Target Retrieval Buffer (ACDBio) at 95–97 °C for 15 min. Slides were washed in distilled water for 15 s and dehydrated in 100% ethanol for 30 s. After air drying, a hydrophobic barrier was drawn using ImmEdge barrier pen and sections were incubated with Protease Plus (both from ACDBio) for 30 min at 40 °C. The subsequent hybridization, amplification and detection steps were performed according to the manufacturer's instructions (Multiplex Fluorescent Detection kit v2, ACDBio). Sections were stained with TO-PRO3, mounted with ProLong Gold Antifade Mountant (Thermo Fisher) and stored at 4 °C in the dark. The percentage of F4-80 mRNA-positive interstitial macrophages, CLS macrophages and MGCs also expressing ChAT mRNA was calculated in 5 mice *per* experimental group. The percentage of CD68 mRNA-positive interstitial macrophages, CLS macrophages, and MGCs also expressing ChAT mRNA was calculated in omental fat sections from 7 obese subjects. For both mouse and human morphometry, three representative 50-μm-spaced sections from each sample were analyzed; from each section, 10 non-overlapping images were randomly selected using a × 63 objective. To quantify mRNA staining, the number of fluorescent dots *per* cell was counted in 26 ChAT mRNA-positive interstitial macrophages from 5 CD mice and in 73 ChAT mRNA-positive interstitial macrophages from 5 HFD mice [35,36]. Co-detection of ChAT mRNA and protein was performed using RNA-protein Co-detection Ancillary kit no. 323180 (ACDBio) according to the manufacturer's instructions.

### 2.9 Liquid chromatography/tandem mass spectrometry

For ACh and choline quantification, eWAT isolated from 25 CD and 25 HFD male mice was weighed and washed 3 times with Hanks' Balanced Salt Solution (HBSS; Sigma–Aldrich) supplemented with 150 μM rivastigmine. For each experimental condition, eWAT from 5 animals was pooled, placed into a Petri dish, and minced with sterile scissors. Tissue fragments were digested with a type II collagenase solution (2 mg/ml in HBSS; Sigma–Aldrich) supplemented with 150 μM rivastigmine for 45 min in a 37 °C water bath, filtered through a 100-μm cell strainer and then centrifuged at 200 rpm for 10 min at RT. Floating cells corresponding to mature adipocytes were discarded, whereas cells of the stromal vascular fraction (SVF) were washed twice with PBS containing 150 μM rivastigmine, incubated with erythrocyte lysis buffer (155 mM NH_4_Cl, 5.7 mM K_2_HPO_4_, 0.1 mM EDTA, pH 7.3) supplemented with 150 μM rivastigmine, and then centrifuged at 200 rpm for 10 min at RT. The cell pellet was resuspended with a mixture of MeOH/H_2_O (80:20 v/v) containing 1 ng/μl choline bromide-(methyl-13C), used as the internal standard (200 ng/sample), and centrifuged twice at 12,000 rpm for 10 min at 4 °C to remove cellular debris. The lysate was dried under N_2_ flow and resuspended in 5 mM NH_4_HCOO in H_2_O (pH 7.5) before injection into the mass spectrometer. ACh and choline were quantified with an LC-MS-MS method using a Luna 5 μm HILIC 200 Å LC column (150 × 3 mm). The mobile phases for the analysis were phase A, 5 mM NH_4_HCOO in H_2_O (pH 7.5) and phase B, MeOH. The gradient was T0 5% A, T21 min 5% A, T5 min 95% A, T5.1 min 95% A, T7 min 5% A, T15 min 5% A with a flow rate of 500 μl/min. Data were acquired on a Sciex Triple Quad 3500 with an HPLC system and a built-in autosampler (all from AB Sciex, Milano, Italy). MultiQuant software (v. 3.0.2; AB Sciex) was used for data analysis and peak review of chromatograms. ACh and choline were quantified using a standard curve prepared with pure standards. Data were normalized to tissue weight expressed in grams (g).

### 2.10 Statistical analysis

All experiments were performed in triplicate. Results are reported as mean ± standard error of the mean (SEM). Comparisons between and among groups were performed respectively with Student's t-test and one-way analysis of variance (ANOVA) followed by Tukey's multiple comparisons post-hoc test. A P-value < 0.05 was considered significant. All statistical analyses were performed with GraphPad Prism 8 software.

## 3 Results

### 3.1 In mouse epididymal WAT, diet-induced obesity affects the expression of cholinergic molecular components

**Figure 1:**
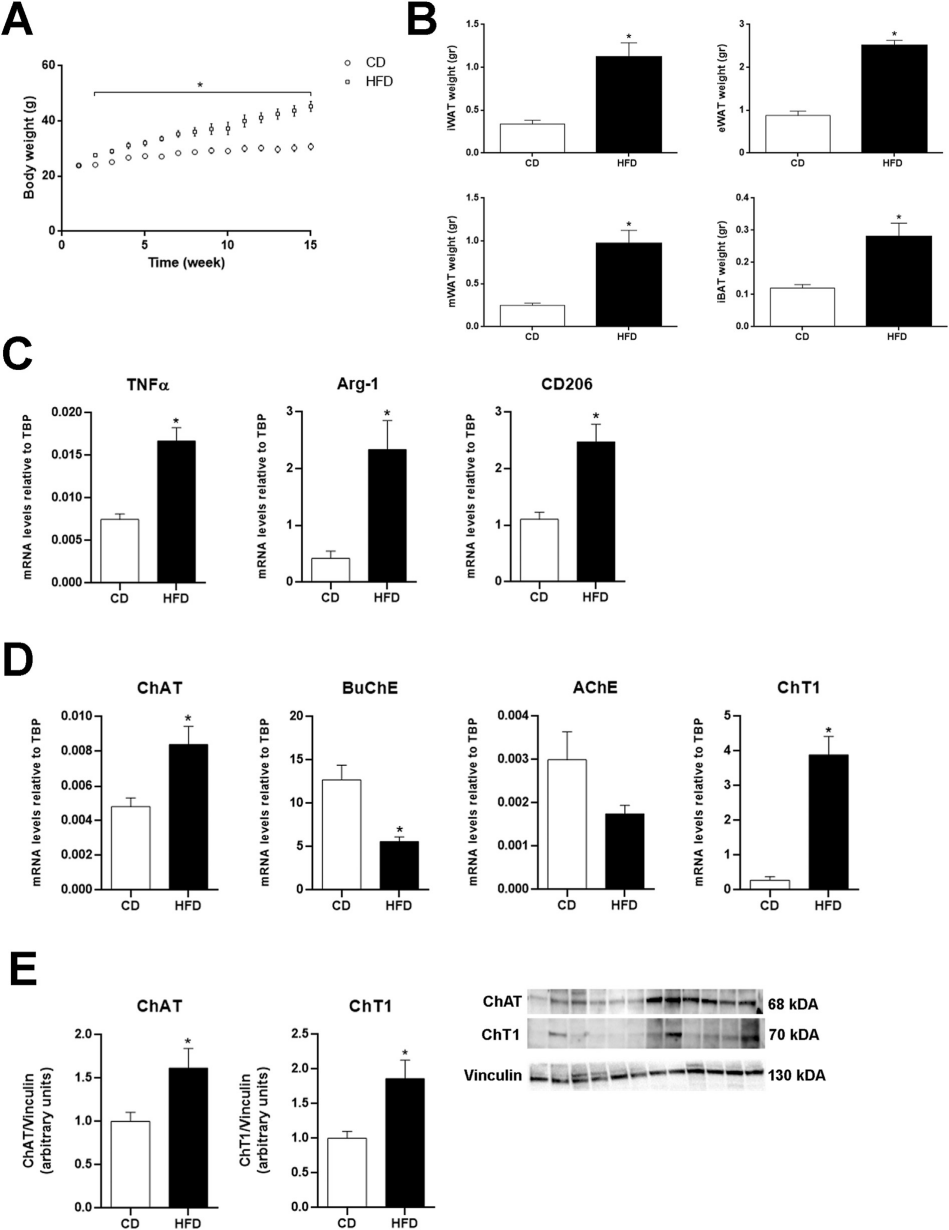
Body weight trends in mice fed a low-fat diet (CD) and an HFD for 15 weeks (A). Visceral and subcutaneous adipose depot weights at the end of the experiment (B). IWAT, inguinal subcutaneous WAT; mWAT, mesenteric WAT; IBAT, interscapular brown adipose tissue. qRT-PCR analysis of macrophage markers (C) and cholinergic molecular components (D) in eWAT from CD (n = 6) and HFD mice (n = 6). E: representative immunoblots and quantification of ChAT and ChT1 in eWAT from CD (n = 6) and HFD mice (n = 6). Data are shown as mean ± SEM; ∗P < 0.05. Data were analyzed using Student's *t-*test.

**Supplementary figure 1:**
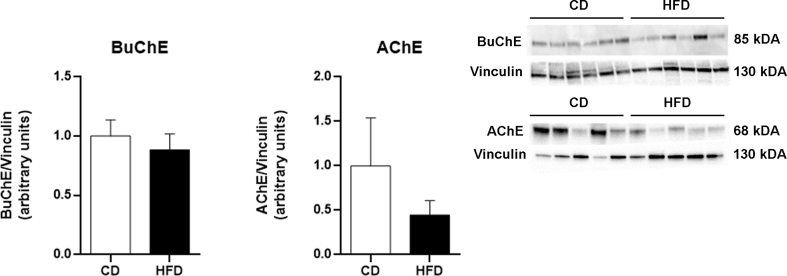
Representative immunoblots and quantification of BuChE and AChE in eWAT from CD (n = 6) and HFD mice (n = 6). Data are shown as mean ± SEM. Data were analyzed using Student's *t-*test.

To assess whether a non-neuronal cholinergic system becomes activated in obese visceral WAT, adult male mice were fed an HFD for 15 weeks. The diet induced a significant increase of body weight (Figure 1A) and of visceral and subcutaneous adipose depot weight (Figure 1B) compared with CD mice. The qRT-PCR results documented a significantly higher eWAT expression of TNFα, a typical proinflammatory M1 macrophage cytokine, and of arginase-1 (Arg-1) and mannose receptor-1 (CD206), two commonly used anti-inflammatory M2 macrophage markers [37], in HFD compared with CD mice (Figure 1C). These data confirmed that the HFD determined significant macrophage infiltration of this visceral WAT depot. Evaluation of the expression of the cholinergic molecular components of eWAT disclosed a significantly increased expression of ChAT and ChT1 mRNAs in obese compared with control mice that was paralleled by a reduced expression of BuChE and AChE mRNAs (although for the latter marker the difference was not significant; Figure 1D). Neither control nor obese mice showed detectable VAChT mRNA (data not shown). Western blot analysis confirmed the significantly increased ChAT and ChT1 expression found in obese mouse eWAT (Figure 1E). However, in obese mouse eWAT the BuChE protein levels were similar to those measured in CD mice, whereas AChE protein merely showed a non-significant reduction (Suppl. Fig. 1). Collectively, these data show that HFD induces eWAT molecular changes that are consistent with increased ACh synthesis, probably in parallel with its reduced degradation. They also suggest that obesity enhances or promotes the activation of cholinergic signaling in this fat depot. The lack of VAChT mRNA in eWAT is consistent with the absence of cholinergic nerves in mouse eWAT [26]. Yet, this does not exclude the presence and/or the activation of a non-neuronal cholinergic system, where ACh is often found not in vesicles, but rather synthesized at very low level in the cytosol for direct release when needed [[38], [39], [40]], possibly through the organic cation transporter [41].

### 3.2 Macrophages infiltrating mouse epididymal WAT express ChAT protein

**Figure 2:**
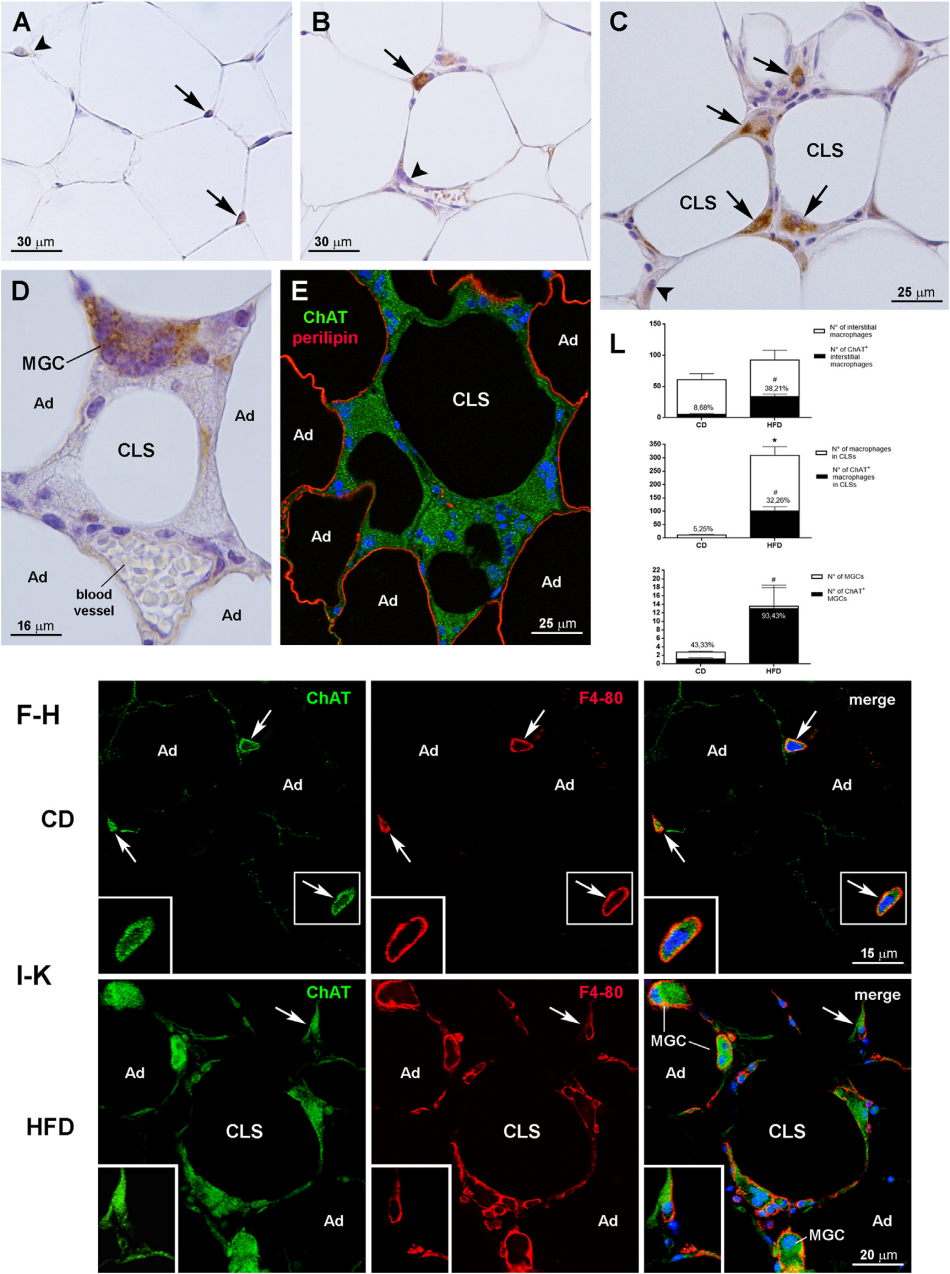
Immunohistochemical detection of ChAT in mouse eWAT from CD and HFD mice. Immunoperoxidase: in CD mice, ChAT staining is barely detectable in two non-adipose cells found among white adipocytes (A, arrows), whereas in HFD mice ChAT is clearly expressed by an infiltrating cell located among the hypertrophic adipocytes (B, arrow), by some cells forming CLSs (C, arrows) and by an MGC (D). Arrowheads in A, B and C indicate negative interstitial cells. By double-staining and confocal microscopy (E), perilipin-positive adipocytes (red) from an HFD mouse do not express ChAT; note the absence of perilipin staining in the CLS depicted in the image, which is formed by intensely ChAT-positive cells (green). Confocal microscopy: in a CD mouse (F–H), three ChAT-positive infiltrating cells (green, arrows) are also positive for the pan-macrophage marker cell surface F4-80 (red, arrows); the inset is the enlargement of the framed area. In an HFD mouse (I–K) specific ChAT staining (green) is detected in an F4-80-positive infiltrating cell (red, arrow) and in F4-80-positive macrophages forming a CLS and three MGCs; the cell on the upper right corner is enlarged in the inset. Ad, adipocyte. L: percentages of F4-80-positive interstitial macrophages, macrophages forming CLSs and MGCs that are also positive for ChAT in eWAT of CD (n = 5) and HFD mice (n = 5). Data are shown as mean ± SEM; ∗P < 0.05 comparing F4-80-positive cells in CD *vs* HFD mice, ^#^P < 0.05 comparing F4-80-positive cells also positive for ChAT in CD *vs* HFD mice. Data were analyzed using Student's *t-*test.

**Supplementary figure 2:**
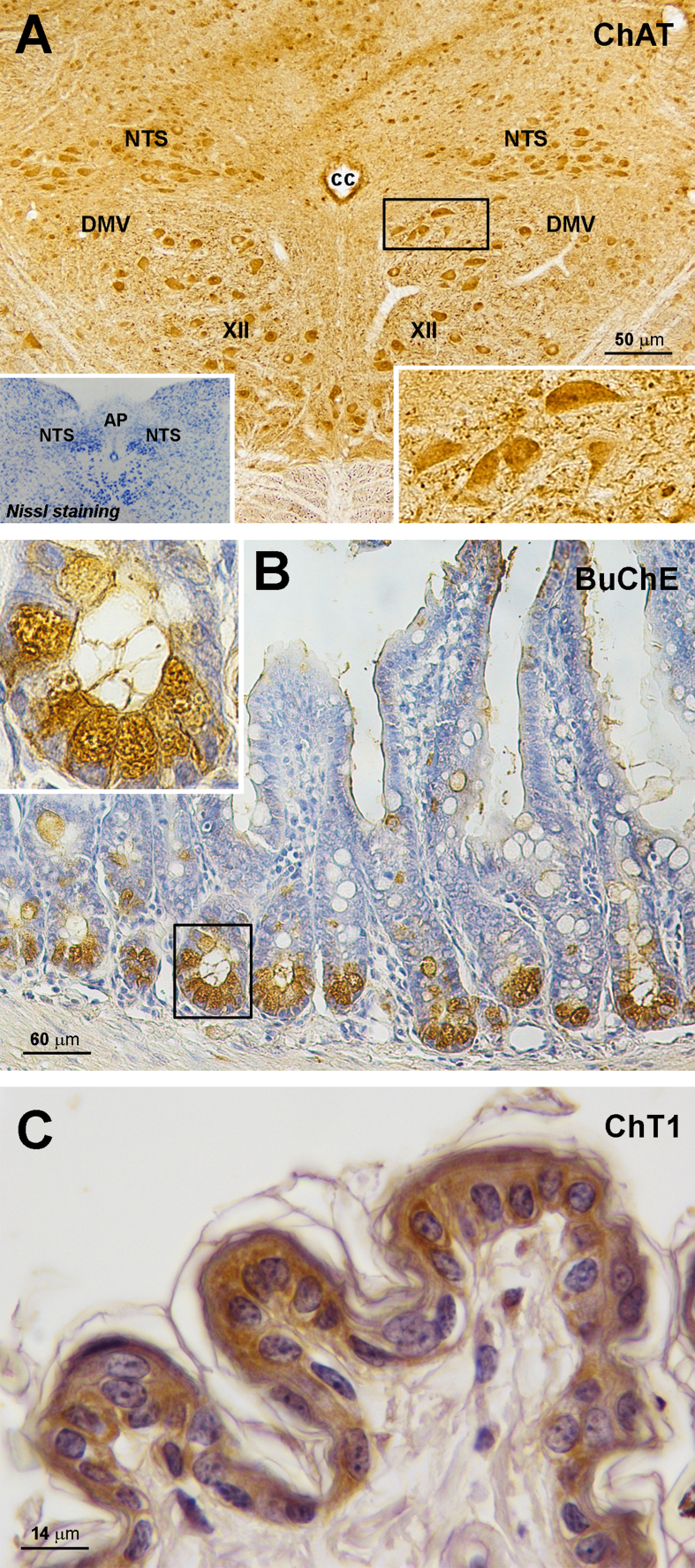
Positive control tissues for the primary antibodies used in the study. A: in a coronal section of the mouse hindbrain, ChAT-positive cholinergic neurons are detected in the solitary tract nucleus (NTS), the dorsal motor nucleus of the vagus nerve (DMV), and the hypoglossal motor nucleus (XII). AP, area postrema; cc, central canal. The inset on the bottom left corner shows an adjacent Nissl-stained section; the inset on the bottom right corner is the enlargement of the framed area. B: Paraffin-embedded gut section showing BuChE-positive Paneth cells in intestinal crypts. The inset is the enlargement of the framed area. C: Paraffin-embedded skin section showing ChT1-positive epidermal cells.

**Supplementary figure 3:**
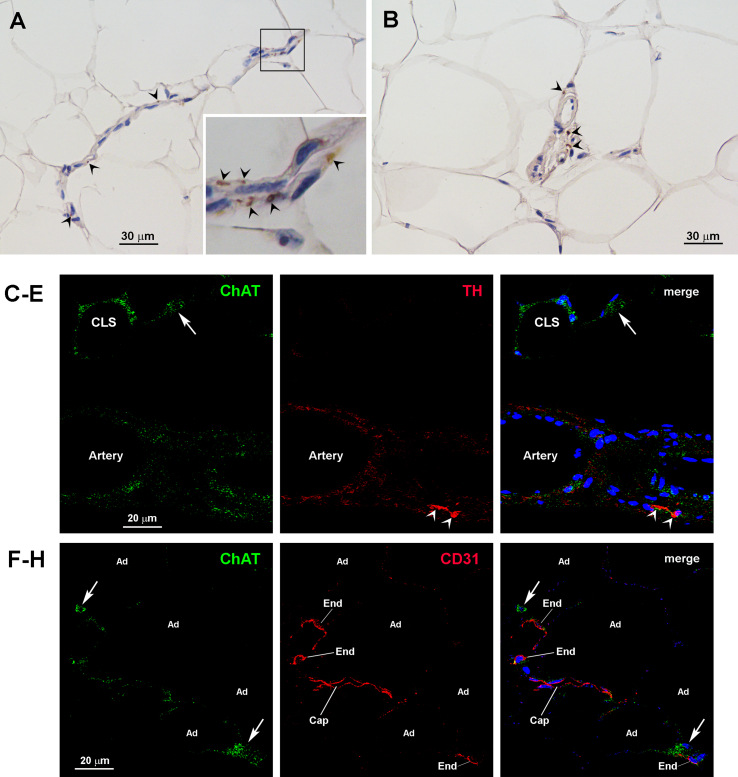
Spatial relationships between ChAT-positive cells and sympathetic noradrenergic nerves and adipose capillaries. By peroxidase immunohistochemistry, tyrosine hydroxylase (TH)-positive noradrenergic nerves (arrowheads) are found around intralobular arteries supplying eWAT in both a CD (A) and an HFD mouse (B). Note the lower number of noradrenergic nerves in the HFD mouse. Inset in A: enlargement of the corresponding framed area. By double staining and confocal microscopy (C–H), ChAT-positive cells from an HFD mouse (C and F, green, arrows) are found at a distance from the vascular noradrenergic nerves (D, red, arrowheads) and in the vicinity of adipocytes (Ad) and CD31-positive parenchymal capillaries (F, red, Cap) or endothelial cells (End). In C-E, the artery wall exhibits non-specific autofluorescence. Ad, adipocyte.

**Supplementary figure 4:**
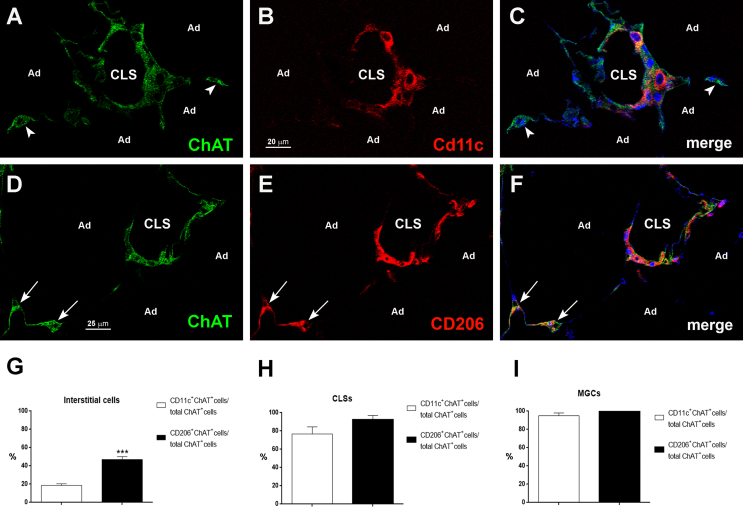
Representative images from the eWAT of an HFD mouse showing colocalization of ChAT-positive cells (A and D, green) and Cd11c- (B, red) or CD206-positive macrophages (E, red) forming CLSs or located among the adipocytes (arrows in D-F). Note that two ChAT-positive cells (arrowheads) in the upper panels are not positive for the M1 macrophage marker Cd11c. Ad, adipocyte. Percentage of ChAT-positive interstitial cells, CLSs and MGCs also expressing the CD11c M1 proinflammatory macrophage marker (white bars) or the CD206 M2 anti-inflammatory macrophage marker (black bars) in eWAT of HFD mice (n = 5). Data are shown as mean ± SEM; ∗∗∗P < 0.001. Data were analyzed using Student's *t-*test.

**Supplementary figure 5:**
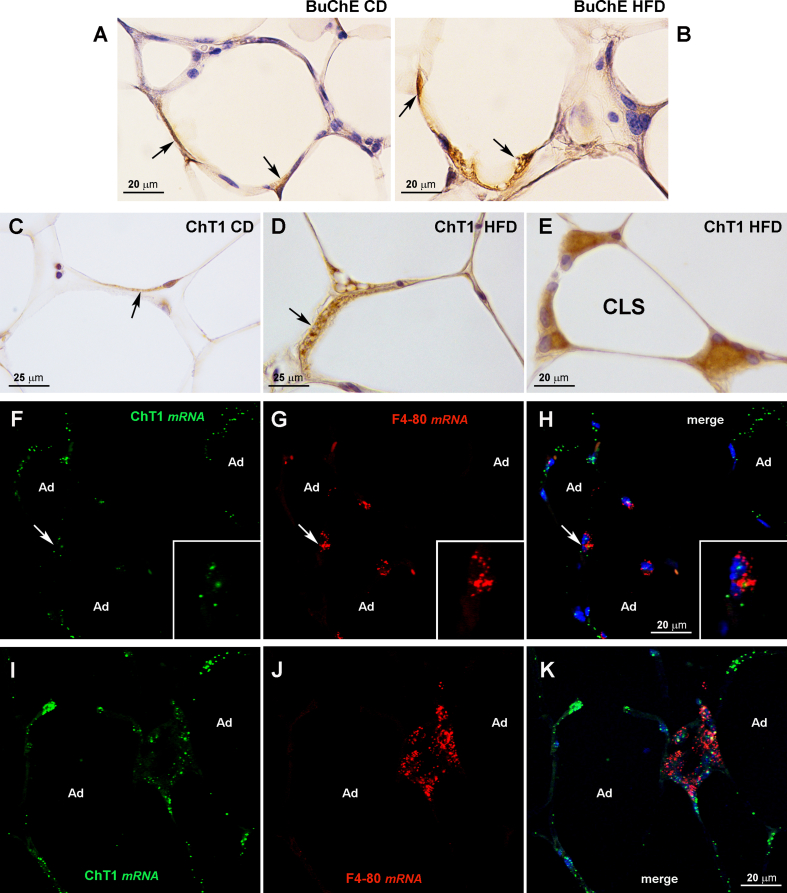
BuChE and ChT1 expression in mouse eWAT from CD and HFD mice. Immunoperoxidase: BuChE immunoreactivity is detected along limited portions of the cytoplasmic rim of white adipocytes (arrows) both in CD (A) and HFD mice (B). In a CD mouse (C), ChT1 immunostaining is barely detectable in the cytoplasmic rim of an adipocyte (arrow); in striking contrast, in an HFD mouse ChT1 staining is strongly expressed by hypertrophic adipocytes (D, arrow) and by macrophages forming a CLS (E). RNA *in situ* hybridization: in a CD mouse (F–H), a small amount of ChT1 mRNA is found in some adipocytes and a macrophages (arrow; enlarged in the inset), whereas in an HFD mouse (I–K) ChT1 mRNA is more abundant in white adipocytes and in macrophages containing F4-80 mRNA. Ad, adipocyte.

To identify the cells involved in this putative adipose non-neuronal cholinergic system, we first employed immunohistochemical techniques to assess the cellular expression and tissue distribution of the cholinergic molecular components. The positive controls for the primary antibodies used in mouse tissue are reported in Supplementary Fig. 2 [42,43]. Histologically, eWAT from CD mice displayed the morphology of normal white fat, whereas eWAT from HFD mice exhibited the typical signature of inflamed obese fat, including several CLSs scattered in the fat lobules and among the hypertrophic adipocytes, a substantial number of MGCs, which were often found near CLSs, and increased cellularity not only in the parenchyma but also in peri- or paravascular position (likely infiltrating inflammatory cells). In CD mice, ChAT immunostaining was barely detectable and then only in a small number of non-adipose cells, which often clustered among white adipocytes (Figure 2A), and in the rare CLSs and MGCs. In obese mice ChAT was expressed by a significant number of infiltrating cells found among the hypertrophic adipocytes (Figure 2B), by numerous cells forming CLSs (Figure 2C), and by several MGCs (Figure 2D). Immunoperoxidase (Figure 2A–D) and immunofluorescence experiments (Figure 2E) showed that white adipocytes never expressed ChAT in either mouse group. Double-staining experiments and confocal microscopy analysis disclosed that in CD mice virtually all ChAT-positive cells infiltrating the adipose parenchyma were also positive for the pan-macrophage cell surface marker F4-80 [44] (Figure 2F–H), thus identifying them as resident interstitial macrophages. In striking contrast, in HFD obese mice only 67.79% ± 4.37 of ChAT-positive interstitial cells were F4-80-positive macrophages (Figure 2I–K), lending support to the possibility that in obese fat other infiltrating cells besides macrophages acquire the ability to synthesize ACh. Morphometric analyses also showed that in CD mice 8.68% ± 3.88 of F4-80-positive interstitial macrophages, 5.25% ± 2.48 of CLS-associated F4-80-positive macrophages, and 43.33% ± 6.66 of F4-80-positive MGCs were also positive for ChAT, whereas in HFD mice ChAT expression was detected in 38.21% ± 3.04 of F4-80-positive interstitial macrophages, in 32.26% ± 1.65 of CLS-associated F4-80-positive macrophages, and in 93.43% ± 4.69 of F4-80-positive MGCs (Figure 2L). To achieve a better characterization of the location of ChAT-positive cells, double-labeling experiments were also performed with tyrosine hydroxylase, a marker of sympathetic noradrenergic nerves, and CD31, an endothelial cell marker. As reported previously [45], noradrenergic innervation in eWAT was sparse, especially in HFD mice, and limited to extralobular and intralobular arteries (Suppl. Figs. 3A and B). In contrast, ChAT-positive cells were always located in the parenchyma, at a distance from arterial vessels and sympathetic noradrenergic nerves (Suppl. Figs. 3C–E), and close to adipocytes and CD31-positive endothelial cells of parenchymal capillaries (Suppl. Figs. 3F–H). Since in morbid obesity WAT is infiltrated by circulating macrophages that display an M1-polarized proinflammatory phenotype [10,37], we evaluated the percentage of ChAT-positive cells also expressing the CD11c M1 proinflammatory macrophage marker or the CD206 M2 anti-inflammatory macrophage marker. In obese mouse eWAT we found similar percentages of ChAT-positive M1 and M2 macrophages forming CLSs and MGCs, whereas interstitial macrophages positive for ChAT largely displayed the M2 anti-inflammatory phenotype (Suppl. Fig. 4). While we were unable to find suitable primary antibodies to detect AChE by immunohistochemistry, we found a similar BuChE expression in adipocytes from both CD and HFD mice, without clear differences between the two conditions, in line with the western blotting data (Suppl. Figs. 5A and B). In striking contrast, whereas ChT1 was poorly expressed by some adipocytes from CD mice (Suppl. Fig. 5C), it was strongly expressed by hypertrophic adipocytes and by numerous macrophages from HFD mice (Suppl. Figs. 5D and E). These findings confirmed the markedly increased expression of this cholinergic marker identified in obese mice by qRT-PCR and western blot analysis.

### 3.3 Macrophages infiltrating mouse epididymal WAT express ChAT mRNA

**Figure 3:**
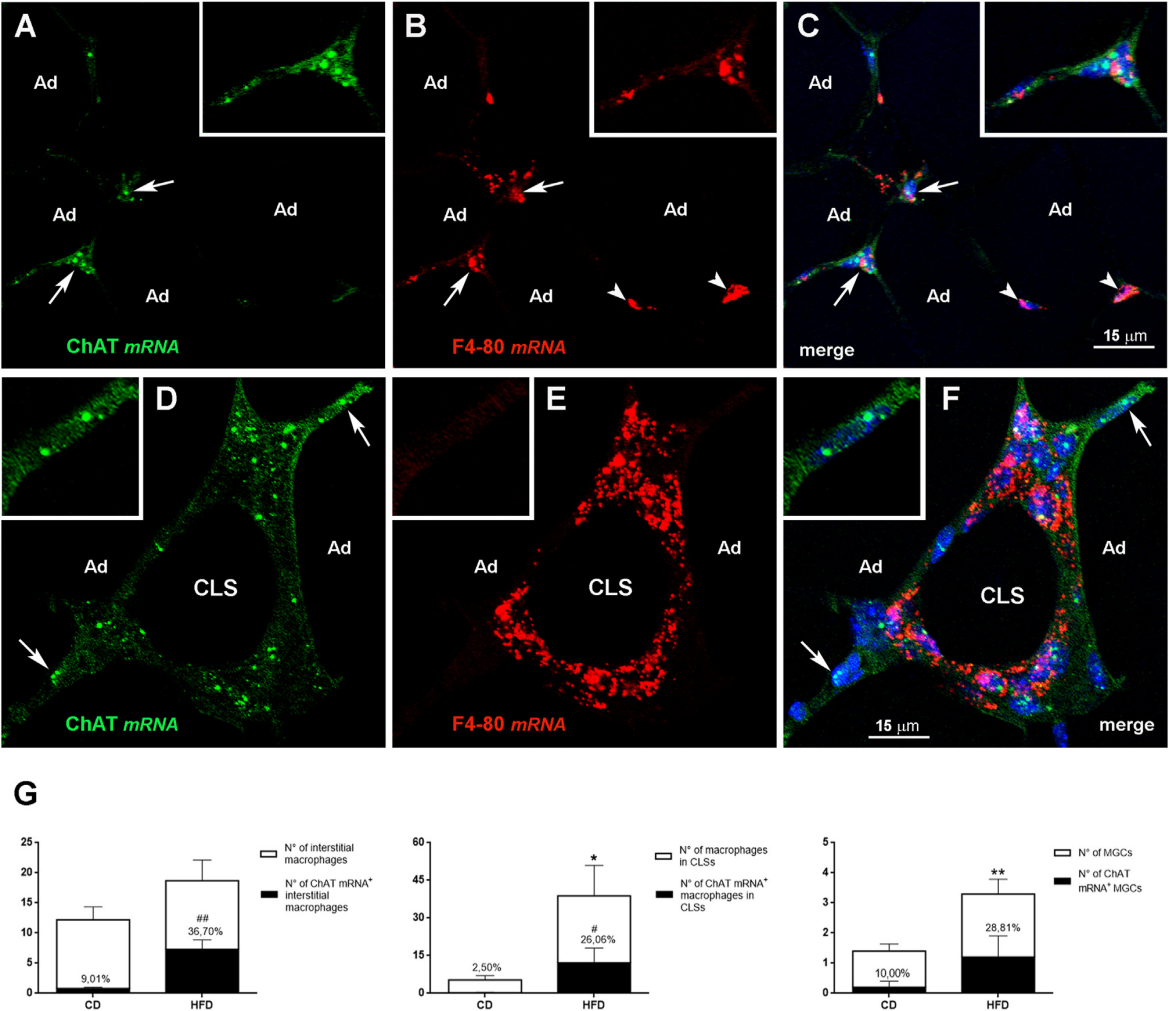
Detection of ChAT mRNA in eWAT from CD and HFD mice by *in situ* hybridization. In a CD mouse (A–C), ChAT mRNA (green) is detected in two interstitial cells also containing F4-80 mRNA (red, arrows); the macrophage found on the bottom left corner is enlarged in the inset. Note the two interstitial cells containing F4-80 mRNA (B, red, arrowheads) and lacking ChAT mRNA. In HFD mice (D–F), ChAT mRNA (green) is detected in several F4-80-positive macrophages forming a CLS (red). Note the two infiltrating cells expressing ChAT mRNA (green, arrows) but containing no F4-80 mRNA. The cell on the upper right corner is enlarged in the inset. Ad, adipocyte. G: percentages of F4-80 mRNA-positive interstitial macrophages, macrophages forming CLSs and MGCs that are also positive for ChAT mRNA in eWAT of CD (n = 5) and HFD mice (n = 5). Data are shown as mean ± SEM; ∗P < 0.05, ∗∗P < 0.01 comparing F4-80-positive cells in CD *vs* HFD mice, ^#^P < 0.05, ^##^P < 0.01 comparing F4-80-positive cells also positive for ChAT in CD *vs* HFD mice. Data were analyzed using Student's *t-*test.

We sought further confirmation that some non-adipose cells in eWAT express ChAT – and are thus potentially capable of synthesizing ACh – by evaluating ChAT mRNA expression and distribution in eWAT from CD and HFD mice with RNA *in situ* hybridization. Whereas in CD mice ChAT mRNA was only detected in a small number of F4-80-positive interstitial macrophages (Figure 3A–C), in HFD mice it was expressed by a substantial number of F4-80-positive interstitial macrophages or macrophages forming CLSs or MGCs (Figure 3D–F). In line with the immunohistochemical experiments, in obese mouse eWAT only 60.89% ± 4.91 of cells found among the hypertrophic adipocytes and expressing ChAT mRNA were also positive for F4-80 mRNA, whereas some cells found among the hypertrophic adipocytes were positive for ChAT mRNA but not for F4-80 mRNA (inset of Figure 3D–F). In addition, in CD mice 9.01% ± 4.20 of F4-80-positive interstitial macrophages, 2.50% ± 2.51 of CLS-associated F4-80-positive macrophages, and 10.00% ± 10.00 of F4-80-positive MGCs were also positive for ChAT, whereas in HFD mice ChAT expression was detected in 36.70% ± 4.62 of F4-80-positive interstitial macrophages, in 26.06% ± 6.40 of CLS-associated F4-80-positive macrophages, and in 28.81% ± 15.20 of F4-80-positive MGCs (Figure 3G). The *in situ* hybridization experiments also confirmed that ChT1 expression in macrophages and hypertrophic adipocytes was higher in obese than in CD mice (Suppl. Figs. 5F–K).

### 3.4 Epididymal WAT from obese mice contains higher levels of acetylcholine and choline

**Figure 4:**
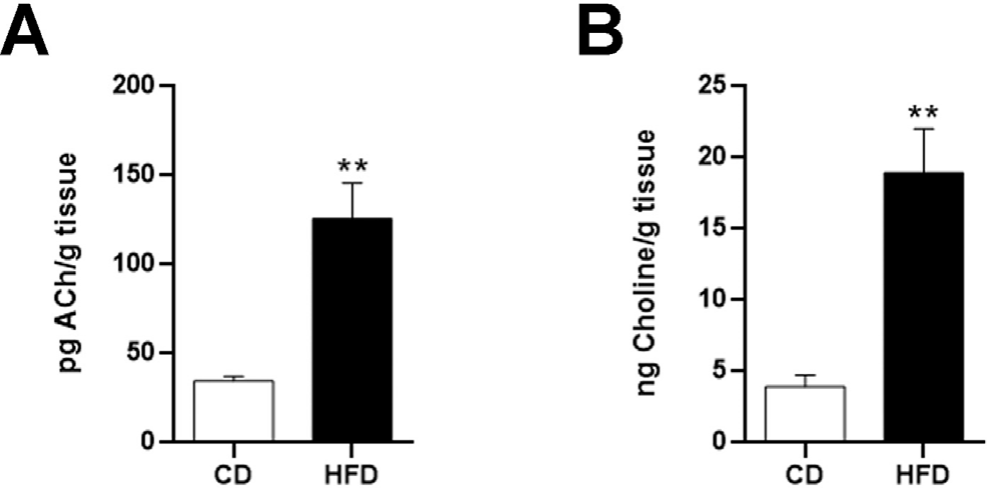
LC-MS-MS analysis of the SVF obtained from eWAT of CD and HFD mice. ACh (A) and choline (B) levels were measured in four SVF samples *per* experimental condition; each sample was obtained by pooling eWAT from five mice. Data are shown as mean ± SEM; ∗∗P < 0.01. Data were analyzed using Student's *t-*test.

Next, we quantified ACh and choline in eWAT SVF from CD and HFD mice using LC-MS-MS. Adipose tissue SVF does not contain fully differentiated adipocytes but is rich in endothelial, interstitial, progenitor and, importantly, infiltrating immune cells [46]. Results normalized for tissue weight (g) demonstrated ACh and choline in the SVF of CD mice (34.75 ± 2.479 pg/g and 3.95 ± 0.81 ng/g, respectively). However, in HFD mouse SVF, ACh was almost 4 times greater (125.78 ± 20.17 pg/g, p < 0.05) (Figure 4A) and choline was about 4.7 times higher (18.95 ± 3.072 ng/g, p < 0.05) (Figure 4B). To determine whether the increased ACh and choline levels seen in obese mice depended on increased ACh production *per* macrophage, due to increased synthesis by ChAT, we performed a semiquantitative evaluation on ChAT mRNA expression [35,36]. The analysis was limited to interstitial macrophages infiltrating eWAT, because CLS-associated macrophages and macrophages forming MGCs did not exhibit clearly recognizable cell boundaries. The fluorescent dots *per* interstitial macrophage were 4 ± 0.47 in CD mice and 4.32 ± 0.33 in HFD mice (p > 0.05). Thus, obesity involved only a modest and not significantly higher expression of ChAT mRNA *per* macrophage, suggesting that the higher ACh and choline levels seen in the SVF of HFD mice are mainly due to the increased number of ChAT-expressing macrophages that infiltrate obese eWAT, as shown in Figure 2, Figure 3G. Moreover, according to the semiquantitative histological scoring method provided by ACDBio for RNAscope® (https://acdbio.com/image-analysis), ChAT staining of macrophages from CD and HFD mice was low to moderate (score 2). Collectively, these data show that mouse eWAT contains a non-neuronal cholinergic system. In healthy mice the system plays as yet unidentified homeostatic functions, but it is importantly recruited and activated in morbid obesity.

### 3.5 Omental fat from obese subjects contains macrophages expressing ChAT protein and mRNA

**Figure 5:**
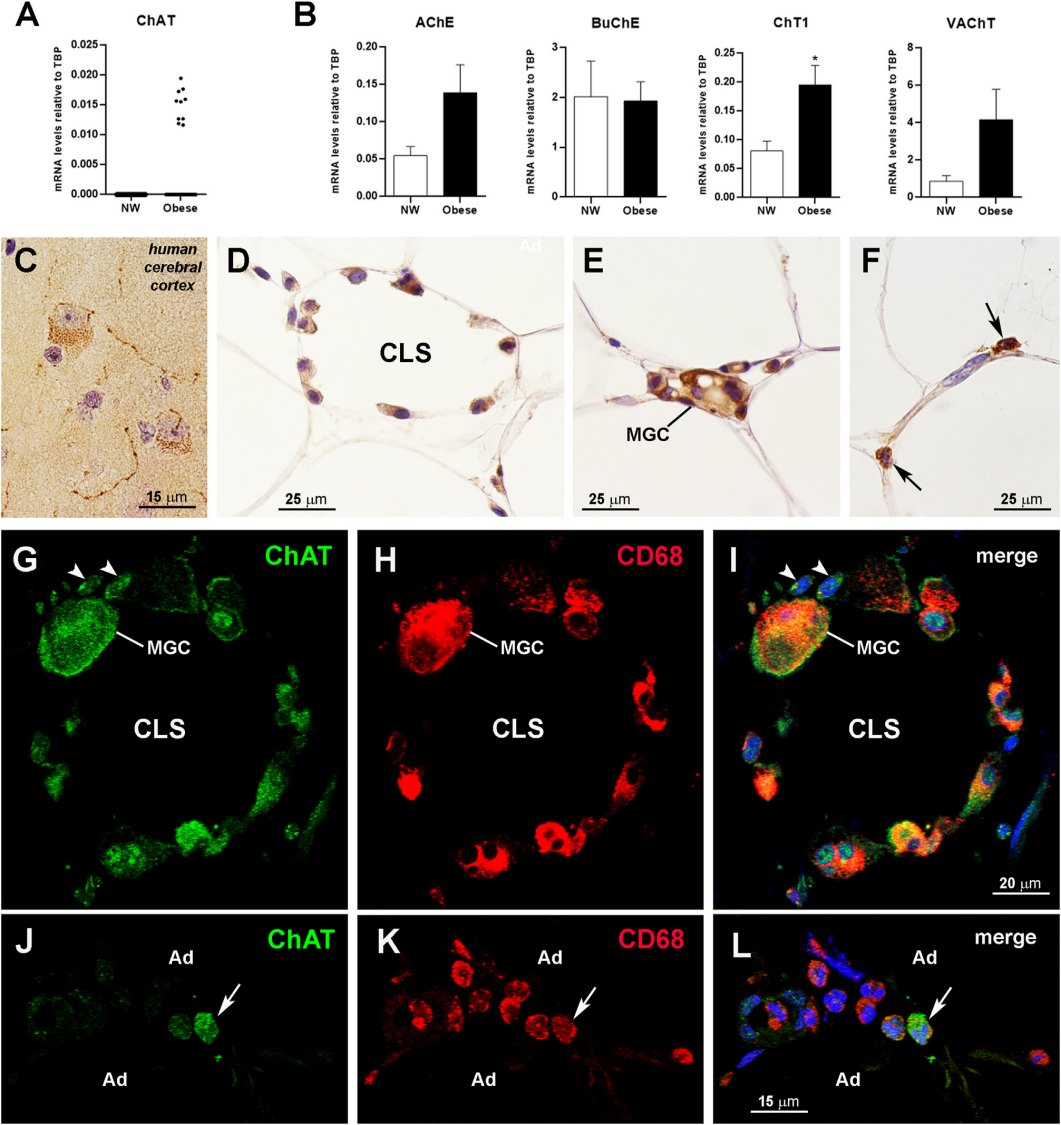
Presence of a non-neuronal cholinergic system in human obese fat. qRT-PCR analysis of ChAT (A) and the other cholinergic molecular components (B) in omental WAT from normal-weight (NW; n = 10) individuals and subjects with obesity (n = 31). Data are shown as mean ± SEM; ∗P < 0.05. Data were analyzed using Student's *t-*test. Immunoperoxidase: specific ChAT staining is detected in neuronal somata and projections of human cerebral cortex (C), used as a positive control; in omental fat from obese patients, ChAT staining is found in macrophages forming a CLS (D) and an MGC (E) and in two infiltrating cells (F, arrows). Double-staining and confocal microscopy (G–I): ChAT-positive macrophages forming a CLS and an MGC (green) are also positive for the macrophage marker CD68 (red). Note two ChAT-positive cells that are however not positive, or very faintly positive for CD68 (arrowheads). J–L: in a small group of CD68-positive macrophages (red) infiltrating omental fat from a subject with obesity, a single cell (green) is also positive for ChAT (arrow). Ad, adipocyte.

**Figure 6:**
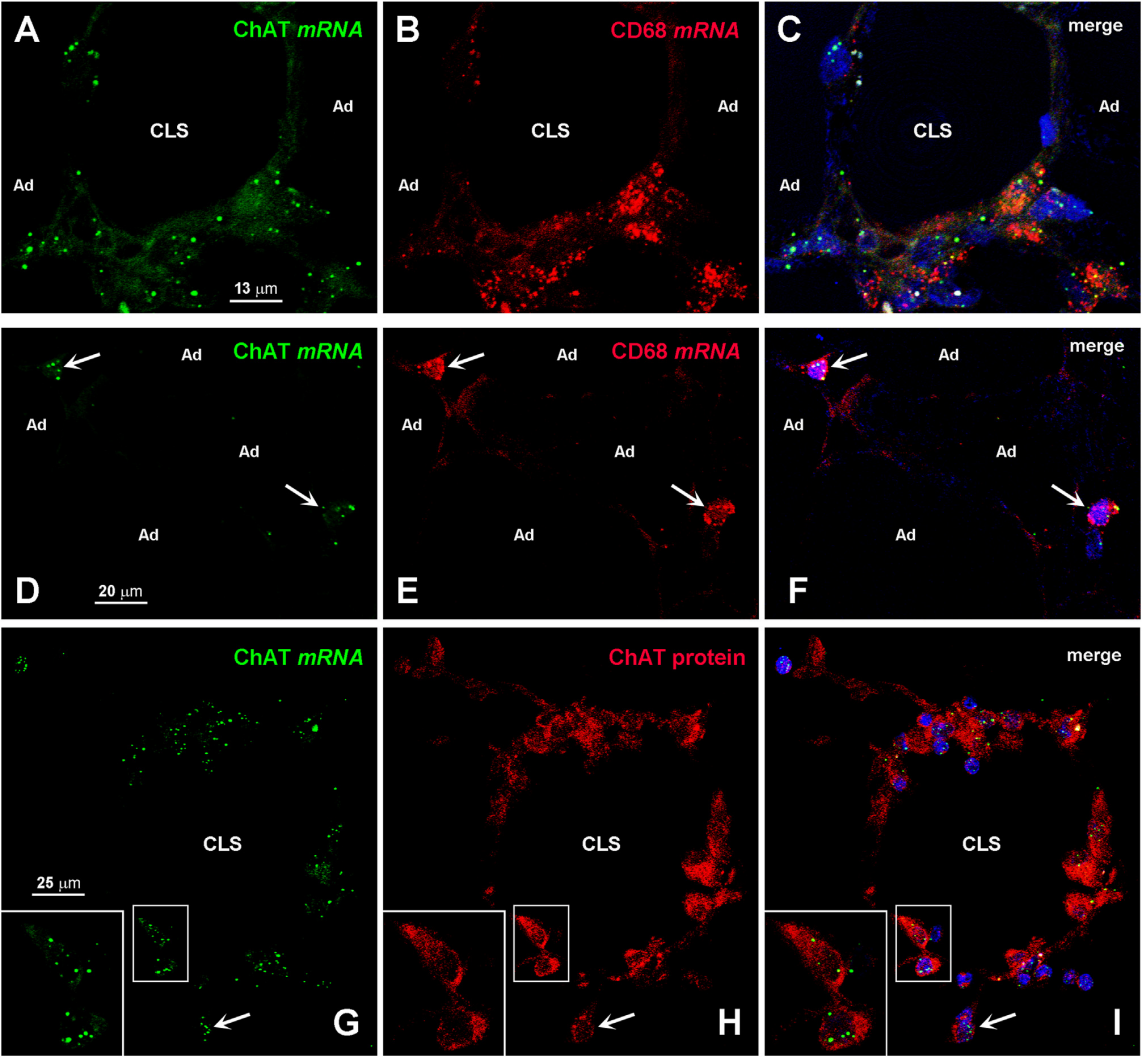
Detection of ChAT mRNA in omental fat from subjects with obesity by *in situ* hybridization. A–C: ChAT mRNA (green) is detected in CD68 mRNA-positive macrophages (red) forming a CLS. D–F: ChAT mRNA (green) is present in two interstitial macrophages (arrows) which are also positive for CD68 mRNA (red). G–I: ChAT mRNA staining colocalizes with ChAT protein staining in macrophages forming a CLS and in two interstitial macrophages (arrows). Insets are enlargements of the corresponding framed areas. Ad, adipocyte.

In a translational perspective, we then assessed whether a non-neuronal cholinergic system could also be detected in human visceral WAT. We first evaluated the cholinergic markers in human omental fat using qRT-PCR. ChAT was undetectable in all healthy subjects (n = 10), whereas it was detected in 10 of the 31 subjects with obesity (Figure 5A). Compared with their healthy counterparts, individuals with obesity showed a higher, albeit not significantly different AChE expression, unchanged BuChE levels and, similar to mouse tissues, significantly increased ChT1 mRNA expression (Figure 5B). Interestingly, and in striking contrast to mouse eWAT, VAChT was detected in omental fat, its expression being slightly but not significantly higher in subjects with obesity (Figure 5B). Although we were unable to find antibodies working properly on paraffin sections to detect the human counterparts of AChE, BuChE, and ChT1 by immunohistochemistry, we found a suitable primary antibody against human ChAT, as demonstrated by specific staining of cholinergic neurons and projections of the human cerebral cortex (Figure 5C). The antibody never yielded specific ChAT staining in omental fat paraffin sections from the healthy subjects. In contrast, it did stain some CD68-positive macrophages forming CLSs (Figure 5D and G-I) or MGCs (Figure 5E and G-I) as well as some interstitial macrophages found among adipocytes (Figure 5F and J-L) in 16 of the 31 subjects with obesity, albeit with considerable variability in the degree of positivity. A retrospective analysis identified ChAT-positive macrophages, CLSs and/or MGCs in 7 of the 10 patients where ChAT had been detected by qRT-PCR and in 9 of the 22 patients where ChAT mRNA was undetectable. Morphometric analysis of omental fat samples containing ChAT-positive cells from 7 obese subjects disclosed that only 64.68% ± 2.37 of ChAT-positive interstitial cells were also positive for the CD68 macrophage marker, suggesting that also in humans other infiltrating cells besides macrophages acquire the ability to synthesize ACh in obesity conditions. In addition, 28.27% ± 2.77 of CD68-positive interstitial macrophages, 19.62% ± 2.26 of CLS-associated CD68-positive macrophages, and 47.61% ± 22.22 of CD68-positive MGCs were also positive for ChAT. No significant correlations were found between the presence and number of ChAT-expressing CD68-positive macrophages and age, gender, or BMI (not shown). Finally, to provide further evidence that macrophages infiltrating obese fat do express ChAT, we resorted again to mRNA *in situ* hybridization. Results showed that whereas no specific staining was detectable in healthy subjects, a number of CD68-positive macrophages forming CLSs (Figure 6A–C) or located among the adipocytes (Figure 6D–F) contained ChAT mRNA. As in mice, the score for ChAT mRNA staining in human macrophages from obese subjects was 2 (from low to moderate) (https://acdbio.com/image-analysis). Co-staining with the ChAT mRNA probe and the ChAT primary antibody yielded an almost complete colocalization of ChAT mRNA and ChAT protein in the same cells (Figure 6G–I). Collectively, these data support the possibility that also in humans – albeit with a high variability that may depend on the anatomical location of the adipose depot, the site of specimen collection, and obesity duration and severity – a macrophage-dependent non-neuronal cholinergic system becomes activated in obese inflamed fat.

### 3.6 Acetylcholine blunts inflammatory gene expression and ameliorates insulin resistance in TNFα-treated human white adipocyte cultures

**Figure 7:**
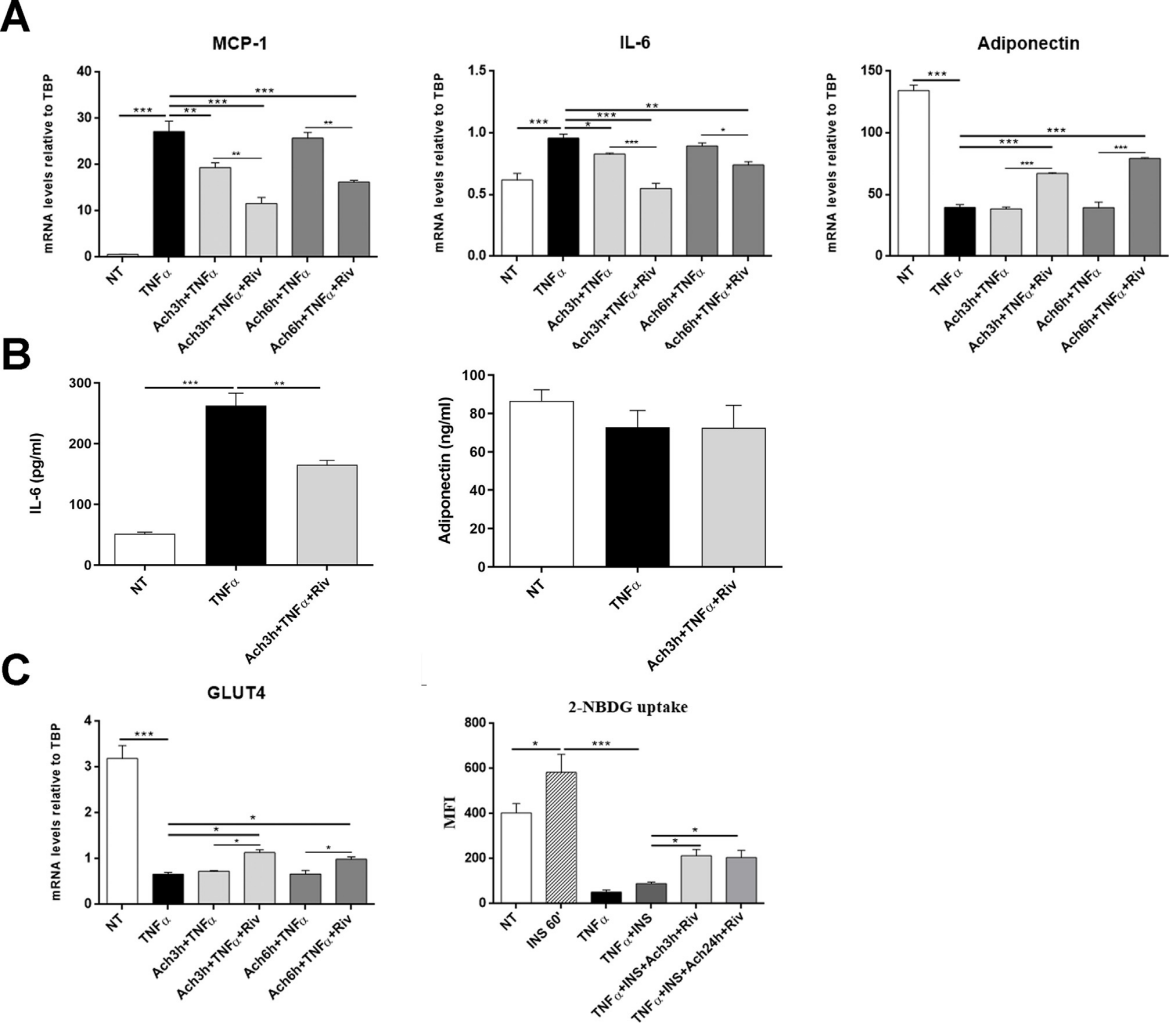
Anti-inflammatory and insulin-sensitizing effects of ACh in hMADS adipocytes. A: MCP-1, IL-6, and adiponectin mRNA levels in hMADS adipocytes treated for 24 h with 10 ng/ml TNFα, alone or combined with 1 μM ACh, with/without 100 nM rivastigmine for 3 or 6 h; ∗P < 0.05, ∗∗P < 0.01, ∗∗∗P < 0.001 between biological groups as indicated by the lines. B: IL-6 and adiponectin concentrations measured by ELISA in the culture supernatants. ∗∗P < 0.01, ∗∗∗P < 0.001 between biological groups as indicated by the lines. C: GLUT4 mRNA levels in hMADS adipocytes treated for 24 h with 10 ng/ml TNFα, alone or combined with 1 μM ACh, with/without 100 nM rivastigmine for 3 or 6 h; ∗P < 0.05, ∗∗∗P < 0.001 between biological groups as indicated by the lines. D: 2-NBDG uptake, expressed as mean fluorescence intensity (MFI), in untreated hMADS adipocytes (NT) and hMADS adipocytes treated with 100 nM insulin for 20 min; 10 ng/ml TNFα for 24 h; with TNFα and insulin; or with TNFα, insulin, 1 μM ACh, and 100 nM rivastigmine for 3 or 24 h ∗P < 0.05, ∗∗∗P < 0.001 between biological groups as indicated by the lines. All experiments were performed in triplicate, data are mean ± SEM and comparisons among groups were performed with ANOVA followed by Tukey's multiple comparisons post-hoc test.

**Supplementary figure 6:**
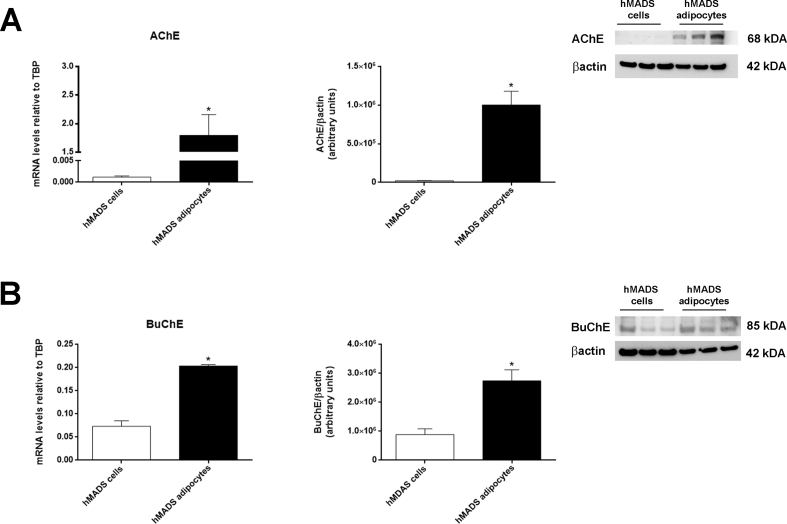
Expression of AChE and BuChE in hMADS adipocytes. qRT-PCR analysis (left-side panels) and representative immunoblots and quantification (right-side panels) of AChE (A) and BuChE expression (B) in hMADS cells (white bars) and hMADS adipocytes (black bars). Data (from 3 samples) are shown as mean ± SEM; ∗P < 0.05 compared with hMADS cells. Data were analyzed using Student's *t-*test.

The findings described above suggest that under obesogenic conditions, mouse and possibly human visceral WAT are infiltrated by a population of ChAT-expressing macrophages that are capable of synthesizing and releasing ACh, which in turn can target adipocytes and affect their metabolism by diffusing in a paracrine way. Thus, we tested some biological effects of ACh on hMADS adipocytes where stress and inflammation had been induced by 24-hour TNFα treatment. As expected, TNFα induced a significantly increased expression of monocyte chemoattractant protein 1 (MCP-1), the main chemotactic factor involved in inflammatory cell infiltration in obese fat [47], and of the proinflammatory cytokine interleukin (IL)-6, whereas it significantly reduced the expression of the insulin-sensitizing hormone adiponectin (Figure 7A). ACh co-treatment for 3 h significantly blunted the effects of TNFα on MCP-1 and IL-6 expression, whereas it did not change adiponectin expression (Figure 7A). However, like most mammalian cells, hMADS adipocytes also express significant amounts of AChE and BuChE, detectable both as mRNA and as protein (Suppl. Fig. 6). Thus, treating TNFα-stressed hMADS adipocytes with ACh and rivastigmine (an AChE and BuChE inhibitor that can prevent ACh degradation) for 3 or 6 h, blunted the effects of TNFα on MCP-1 and IL-6 expression to an even greater extent, while also significantly raising adiponectin mRNA expression (Figure 7A). ELISA, performed in cell culture supernatants, confirmed that treatment with ACh and rivastigmine for 3 h significantly reduced the increased IL-6 secretion in TNFα-stressed hMADS adipocytes but did not change adiponectin secretion (Figure 7B). IL-1β and leptin mRNAs significantly increased in TNFα-stressed hMADS adipocytes, whereas ACh and/or ACh plus rivastigmine did not change their expression (data not shown). In hMADS adipocytes, 24-hour TNFα treatment also reduced GLUT4 mRNA expression, which was not changed by ACh treatment for 3 or 6 h but was significantly restored by ACh and rivastigmine treatment for 3 or 6 h (Figure 7C). Finally, 24-hour TNFα treatment drastically reduced hMADS adipocyte glucose uptake, induced by 60-minute insulin treatment, but treatment with ACh and rivastigmine for 3 or 24 h significantly improved glucose uptake (Figure 7D).

## 4 Discussion

In rodents and humans, white adipocytes express a complex, heterogeneous and developmentally regulated pattern of mAChRs and nAChRs that is modulated by a variety of pathophysiological conditions including cold and obesity [[13], [14], [15], [16], [17], [18], [19],^22^,^23^]. However, since WAT lacks parasympathetic cholinergic innervation [26], the ACh acting on adipocytes cannot be of neural origin, but must be synthesized and secreted by non-neuronal WAT cells. Blood is unlikely to be the source of endogenous ACh, since significant cholinesterase activity in plasma, erythrocytes, and liver and lung endothelial cells keeps it at nearly undetectable levels, to prevent any ACh that may spill into the circulation from acting in a diffuse manner, like a hormone [[27], [28], [29]]. Here, we show for the first time that mouse eWAT is provided with a non-neuronal cholinergic system whereby macrophages found in healthy tissue and, importantly, those infiltrating eWAT in individuals with morbid obesity, possess and/or acquire the ability to express ChAT (mRNA and protein), the enzyme driving ACh synthesis, and to synthesize ACh. Interestingly, in obese mice a substantial population of ChAT-positive infiltrating cells were not also positive for F4-80, a glycoprotein that is expressed in a wide range of mature tissue macrophages and is considered as a pan-macrophage marker [44]. A similar pattern of staining was also found in humans, using CD68 as macrophage marker. This raises the interesting possibility that other cell types, such as lymphocytes and/or granulocytes, may be able to synthesize and secrete ACh in inflamed obese WAT. These data, in conjunction with the significantly higher ACh concentration found in the SVF of obese compared with lean mice, highlight the presence of an immune-mediated non-neuronal cholinergic system in eWAT that becomes functionally activated in obesity-induced inflammation.

This investigation was conducted in mouse eWAT for three main reasons. Even though recent single-cell studies have highlighted a limited overlap of human and mouse adipocytes and adipose depots in normal and obese conditions [48,49], the rodent epididymal depot is the most similar to human visceral fat, sharing a high susceptibility to macrophage infiltration and providing a source of inflammatory mediators that greatly contribute to the metabolic complications of obesity [4,5]. Moreover, the rodent eWAT is a large and completely dissectible fat depot, which can be collected without contamination from adjacent organs/structures [50]. Finally, the anatomical organization of mouse eWAT is such that, unlike the mesenteric, parametrial and subcutaneous adipose depots, it is not traversed by motor or parasympathetic cholinergic nerves targeting adjacent somatic or visceral structures. It also contains neither lymph nodes nor permanent lymphatic tissue foci [51], whose expression of molecules involved in cholinergic transmission could interfere with the molecular investigation of cholinergic components.

Our data support the hypothesis that in inflamed obese visceral WAT, a large number of macrophages, CLSs and MGCs – more numerous in mice than in humans – synthesize and secrete ACh, which by diffusing through the extracellular space has the potential to affect the metabolism of adjacent adipocytes. In line with studies conducted with specific AChR agonists in mouse and human cultured adipocytes [[14], [15], [16], [17], [18], [19]], we found that ACh exerted anti-inflammatory effects and promoted insulin sensitivity in hMADS adipocytes stressed by TNFα treatment. Interestingly, rivastigmine blockade of the hydrolytic activity of AChE and BuChE, which are expressed by white adipocytes, enhanced such beneficial effects. While the expression of these enzymes by adipocytes agrees with the notion that they are targeted by ACh, it also suggests that selective enhancement of cholinergic signaling to white adipocytes *via* reduction of AChE and/or BuChE activities could provide a novel therapeutic approach to morbid obesity. The effects of ACh secreted by inflammatory cells in WAT may contribute to the morphological and metabolic heterogeneity of obese fat adipocytes, where normal-appearing adipocytes are frequently detected among stressed and disrupted adipocytes [52]. In addition, obese fat displays remarkable regenerative abilities over time [53]. Whereas a possible action of ACh on adipocyte precursors has not yet been investigated, both rodent and human adipose-derived mesenchymal stem cells express mAChRs and nAChRs, whose activation affects their differentiation fate [54,55]. Of course, ACh produced by inflammatory cells infiltrating obese WAT may also affect non-adipose cells in an autocrine or a paracrine way, for instance by targeting macrophages. Whereas activation of macrophage mAChRs exerts proinflammatory effects [56], promotion of macrophage nAChR signaling is generally associated with an anti-inflammatory action. Specifically, activation of macrophage α7nAChR appears to reduce inflammatory cytokine secretion and to affect apoptosis, proliferation, and macrophage polarization, eventually reducing the systemic inflammatory response [57]. Interestingly, α7nAChR knockout mice challenged with an HFD show greater adipose tissue inflammation and greater impairment of insulin sensitivity [22,58]. Vascular endothelial cells express both mAChRs and nAChRs and respond to ACh by releasing nitric oxide, which promotes vasodilation [59]; in addition, nicotine is a potent stimulator of angiogenesis, mainly mediated by α7nAChR, and involves activation of mitogen-activated protein kinase and transcription factor NF-қB [60]. As a consequence, the non-neuronal cholinergic system identified in our study could be implicated in the important vascular changes detected in obese WAT. Finally, ACh also has the potential to affect adipose tissue fibrosis by acting on fibroblasts [61].

The conventional view is that, in obesogenic conditions, WAT, which normally contains resident macrophages, is populated by circulating macrophages that display an M1-polarized proinflammatory phenotype, which promotes low-grade inflammation and insulin resistance [10,37]. In fact, recent single-cell studies have stressed the wide heterogeneity of the adipose immune cell compartments in normal and obese fat, also questioning the clear-cut distinction between M1 proinflammatory and M2 anti-inflammatory macrophages [62]. Our findings that ChAT was mostly expressed by CD206-positive M2 anti-inflammatory interstitial macrophages may suggest that activation of the non-neuronal cholinergic system takes place in distinct sets of macrophages, possibly those involved in plasticity and repair. However, the embryonic or bone marrow origin of tissue macrophages, their context-dependent expandability through *in situ* proliferation, and their molecular signatures and roles in steady-state or pathological conditions are rapidly evolving fields in immunity research. Thus, as recently described in the heart [63], the WAT macrophage compartment could prove to be rather more heterogenous and dynamic than anticipated. Clearly, studies harnessing a combination of fate mapping, flow cytometry and single cell genomic techniques are needed to establish which macrophage subtype(s) express ChAT and produce ACh in normal and obese visceral WAT.

In humans, obesity did not induce significant changes in omental AChE or BuChE mRNA expression. Human tissues are more complex than mouse tissues, and, importantly, they are characterized by a greater interindividual variability. In addition, these cholinesterases are involved in other ACh-independent metabolic pathways that can modify their expression in tissues. Hopefully, expanding patient number will provide further informative data and possibly even demonstrate a modulation of cholinesterase activities by obesity also in human fat. Interestingly, and in striking contrast to mouse eWAT, VAChT expression was detected in human omental fat (by qRT-PCR) and was increased, albeit not significantly, in subjects with obesity. Even though VAChT is considered as a typical signature of neuronal cholinergic transmission, it has also been detected in some non-neuronal cholinergic systems, where it is expressed by ACh-producing alpha cells of human pancreatic islets [64], ACh-producing human cardiomyocytes [65] and, importantly, ACh-producing human lung macrophages [66]. Accordingly, and at variance with mouse eWAT, VAChT could thus be responsible for ACh storage and secretion in the macrophage-dependent non-neuronal cholinergic system we identified in obese human omental fat. Finally, even if omental VAChT expression were related to the presence of cholinergic nerves in this depot - arising from the parasympathetic system or belonging to the enteric nervous system -, it would not exclude the presence of a non-neuronal cholinergic system. However, our findings seem to rule out significant cholinergic innervation of omental fat since ChAT immunohistochemistry clearly demonstrated cholinergic axonal projections in the human cortex (Figure 5C) but did not demonstrate nerve-like structures in human omental fat.

Further aspects emerging from our study are an increased ChT1 expression in obese fat from both mice and humans and higher choline levels in the SVF of obese mouse eWAT. These findings indicate that choline metabolism is accelerated in obesity. Enhancement of choline metabolism in obese fat may to some extent reflect the stimulation of non-neuronal cholinergic signaling. Interestingly, choline is a partial nAChR agonist, and concentrations greater than those needed to block these ionotropic channels induce their activation [67,68]. Thus, a contribution of choline to the non-neuronal cholinergic system acting in obese fat should not be excluded. On the other hand, some cellular events occurring in obese inflamed fat, including adipocyte hypertrophy and macrophage recruitment, migration and phagocytosis, require substantial *de novo* synthesis of cell membranes, where choline is required for the production of essential membrane phospholipids. Whatever the cause, the increased amount of choline required for WAT plastic adjustments during obesity may reduce its availability to other tissues and organs and compound some pathological consequences of its comorbidities. Interestingly, in humans dietary choline deficiency results in liver and muscle damage, including liver steatosis and elevated serum levels of hepatic transaminases and creatine phosphokinase [69,70].

ACh synthesis and ChAT mRNA and/or protein in macrophages were first reported in human alveolar macrophages [71] and in rat kidney macrophages during acute allograft rejection [72]. Subsequently, the expression of the reporter gene was detected in splenic macrophages of ChAT^BAC^-eGFP transgenic mice [73]. Our finding of a macrophage-dependent non-neuronal cholinergic system in visceral WAT and its activation during obesity is in line with research showing that macrophages secreting ACh regulate thermogenic activation in mouse subcutaneous fat through α2nAChR [23,74]. Critically, this non-neuronal cholinergic system is activated not only by cold exposure but also by high-fat feeding [23,74]. Thus, mice with an adipocyte-specific deletion of α2nAChR show an impaired subcutaneous thermogenic response after HFD, leading to exacerbated obesity and reduced glucose tolerance. These data suggest that in mouse subcutaneous fat HFD activates a non-neuronal cholinergic system that promotes non-shivering thermogenesis-dependent energy expenditure in an attempt to protect the organism against calorie overload and metabolic stress [75]. Consequently, the emerging view is that, as in several other inflammatory conditions, during morbid obesity non-neuronal cholinergic systems become activated and exert beneficial depot-specific effects on distinctive pathophysiological aspects of morbid obesity; these aspects may range from reduction of WAT inflammation and insulin resistance to increase of brown fat-mediated energy expenditure. Although such systems cannot rescue the adipose tissue disruption or the metabolic impairment, they likely represent compensatory responses of the body to the obesogenic condition. Mouse models where white adipocyte cholinergic stimulation is enhanced by, for example, deleting adipocyte cholinesterase activity, will hopefully reveal the pathophysiological relevance of the non-neuronal cholinergic system in visceral fat and its potential to be harnessed to combat morbid obesity and metabolic syndrome. If this were the case, its selective boosting might provide a further therapeutic approach to counteract obesity and associated diseases.

In conclusion, visceral WAT, like other tissues like the spleen, placenta, epidermis, urothelium and airway epithelium [[27], [28], [29]], is provided with cholinergic transmission that exerts important effects in physiological and/or pathophysiological conditions, despite lacking significant parasympathetic cholinergic innervation. Here, we present evidence that non-neuronal ACh may play beneficial effects on white adipocytes. However, ACh is a potent pleiotropic molecule whose receptors are probably found in all cell types. In rodents, but especially in humans, adipose tissue often surrounds and infiltrates organs and tissues including the mammary gland; the pancreas, especially in older individuals; the heart, at the level of the epicardial layer; skeletal muscle; and the main blood vessel and nerve bundles. Thus, if activation of the non-neuronal cholinergic system were confirmed to be a diffuse compensatory response in adipose tissue during morbid obesity, it would be possible to advance the hypothesis that ACh, oversecreted by immune cells infiltrating these obese depots, may also interact with discrete cell types, disrupt local homeostatic mechanisms, and contribute to obesity-related comorbidities such as breast cancer, diabetes, cardiovascular disease and peripheral neuropathy.

## CRediT authorship contribution statement

**Ilenia Severi:** Data curation, Investigation, Writing – original draft, Formal analysis, Methodology. **Jessica Perugini:** Data curation, Investigation, Writing – original draft, Formal analysis. **Chiara Ruocco:** Investigation, Methodology. **Lara Coppi:** Investigation, Methodology. **Silvia Pedretti:** Investigation, Methodology. **Eleonora Di Mercurio:** Investigation, Methodology. **Martina Senzacqua:** Investigation, Methodology. **Maurizio Ragni:** Investigation, Methodology. **Gabriele Imperato:** Investigation, Methodology. **Alessandra Valerio:** Supervision. **Nico Mitro:** Resources, Supervision, Funding acquisition. **Maurizio Crestani:** Resources, Supervision, Funding acquisition. **Enzo Nisoli:** Resources, Supervision, Funding acquisition. **Antonio Giordano:** Conception and design, Data analysis, Funding acquisition, Supervision, Resources, Manuscript writing.

## Declaration of competing interest

The authors declare that they have no competing interests.

## Data Availability

Data will be made available on request.
