# Supplementary material for: Activation of a non-neuronal cholinergic system in visceral white adipose tissue of obese mice and humans
Source: Mol Metab. 2023 Dec 22;79:101862. doi: 10.1016/j.molmet.2023.101862 (PMC10792749; doi:10.1016/j.molmet.2023.101862)
Supplement: Multimedia component 1 [file mmc1.docx]

**TABLE 1A**. Taqman probes all from Applied Biosystems #4453320.

| Target Gene | Assay ID |
| --- | --- |
| AChE  AChE  Adiponectin  Arg-1  BuChE  BuChE  CD206  ChAT  ChAT  ChT1  ChT1  GLUT4 | Hs00241307_m1  Mm00477275_m1  Hs00605917_m1  Mm00475988_m1  Hs00992319_m1  Mm00515326_m1  Mm01329362_m1  Hs00758143_m1  Mm01221880_m1  Hs00222367_m1  Mm00452075_m1  Hs00168966_m1 |
| IL-6 | Hs00174131_m1 |
| MCP-1  TBP  TBP  TNFα | Hs00234140_m1  Hs00427620_m1  Mm00446973_m1  Mm00443258_m1 |
| VAChT  VAChT | Hs00268179_s1  Mm00491465_s1 |

**TABLE 1B**. Primary antibodies.

| Antibodies | Host^*^ | Diluition | Source |
| --- | --- | --- | --- |
| AChE  BuChE  CD11c  CD206  CD31  CD68  ChAT  ChAT  ChT1  F4-80  Perilipin  Tyrosine hydroxylase (TH)  Vinculin | M  G  R  R  R  M  G  R  R  R  R  R  M | 1:1000 (IHC)  1:1000 (WB)  1:1000 (IHC)  1:1000 (WB)  1:400 (IF)  1:800 (IF)  1:200 (IF)  1:250 (IF)  1:600 (IHC)  1:400 (IF)  1:1000 (WB)  1:600 (IHC)  1:1000 (WB)  1:400 (IF)  1:500 (IF)  1:300 (IF)  1:3000 (WB) | Sigma-Aldrich/A6970  R&D Systems/AF-9024  Cell Signaling/97585  Abcam/ab64693  Cell Signaling/D8V9E  Agilent, DAKO/M081401  Merck Millipore/AB144P  Merck Millipore/AB143  Proteintech/21848-1AP  Cell Signaling/70076  Abcam/ab3526  Merk Millipore/AB1542  Sigma-Aldrich/MAB3574 |
|  |  |  |  |

*M, mouse; R, rabbit; G, goat; WB, western blotting; IHC, immunohistochemistry; IF, immunofluorescence.

**TABLE 1C**. Secondary antibodies.

| Conjugated to | React∗ | Dilution | Source | ID |
| --- | --- | --- | --- | --- |
| Peroxidase | M | 1:5000 | Jackson ImmunoResearch | 715-036-150 |
| Peroxidase  Fluorophore  Fluorophore  Biotinylated  Biotinylated  Biotinylated | R  G  R  G  R  M | 1:1000  1:400  1:400  1:200  1:200  1:200 | Vector Laboratories  Invitrogen  Invitrogen  Vector Laboratories  Vector Laboratories  Vector Laboratories | PI-1000  A-11055  A-31572  BA-5000  BA-1000  BA-2000 |

*M, mouse; R, rabbit; G, goat.

**TABLE 1D**. RNAscope Probe all from ACD Bio.

| Target Gene | Assay ID |
| --- | --- |
| CD68  CHAT  ChAT  F4-80  ChT1  3-plex Positive control  3-plex Positive control  3-plex Negative control | Hs-CD68-C2-mRNA (560591-C2)  Hs-CHAT-mRNA (450671)  Mm-ChAT-mRNA (408731)  Mm-Adgre1-C2-mRNA (460651-C2)  Mm-Slc5a7-mRNA (439941)  Hs (PN 320861)  Mm (PN 320881)  Bacterial dapB (PN 320871) |
